# Treatise on Gynecology

**Published:** 1892-06

**Authors:** 


Treatise on Gynecology, Medical and Surgical. By S. Pozzi, M. D., Professor Agrégé á la Faculté de Médecine; Chirurgien de l'Hôpital Lourcine-Pascal, Paris; Honorary Fellow of the American Gynecological Society. Translated from the French edition under the supervision of, and with additions by, Brooks H. Wells, M. D., Lecturer on Gynecology at the New York Polyclinic; Fellow of the New York Obstetrical Society, and the New York Academy of Medicine. Volume II. With 174 wood engravings, and nine full-page plates in color. New York: William Wood & Co. 1892.

The second volume of this admirable treatise is now before us, and we proceed to name some of the special points of its excellence, though we find very little occasion to change our opinion of the general scope and value of the work, which we indicated in our notice of Volume I., in the May issue of the Journal.

Beginning with Inflammation of the Uterine Adnexa, Pozzi first gives the classification, in Chapter 1, of Tubal Inflammations. He next takes up Non-Cystic Salpingitis, which includes the various conditions arising from infections, such as pertain to gonorrhea, parturition, and catarrhal disease. In the next chapter we find Pyosalpinx, Hydrosalpinx, and Hematosalpinx described under the general term of Cystic Oöphoro-Salpingitis. A handsome colored plate is introduced, illustrating a variety of Hematosalpinx simulating Tubal Pregnancy, which was furnished by Dr. Joseph Price. Also one from the same author, illustrating Pyosalpinx and

Ovarian Abscess with Universal Adhesions. These assist very much in the elaboration of this interesting subject, and are a decided addition to the volume. The pathology of pelvic abscess, so-called, is one of the most interesting of all the questions pertaining to pelvic surgery, and much light has lately been thrown upon the subject by a few American operators, who are constantly giving to periodical literature the benefits of their experiences. The editor has shown his appreciation of their work by introducing illustrations of it into this book, and the wisdom of such a course will be appreciated by all who have to deal with these important questions.

Under the title Extra-Uterine Pregnancy will be found some of the opinions of the author which have been developed through recent experience. We need not here enlarge upon this subject, for it has already become one of settled opinion with reference to many points, and in the main our author does not differ essentially from the most modern judgment on the subject. Plastic Surgery of the Genital Tract is considered at length in this volume, as well as Malformations of the Vagina and Uterus, together with Disorders of the Urinary Tract, and Diseases of the Rectum and Pelvis, which latter subject concludes the volume. An index of both volumes is added, which helps to make easy of reference, one of the most interesting and useful treatises upon gynecology which the decade has produced. The perfect press-work and binding are precisely like the first volume, and the two books make a handsome show upon the library shelves.

